# Septic shock and the use of norepinephrine in a high dependency unit - mortality and adverse events

**DOI:** 10.1186/2197-425X-3-S1-A877

**Published:** 2015-10-01

**Authors:** M Hallengren, P Åstrand, S Eksborg, H Barle, C Frostell

**Affiliations:** Karolinska Institutet, Clinical Sciences Danderyd Hospital, Stockholm, Sweden; Karolinska Institutet, Department of Internal Medicine Danderyd Hospital, Stockholm, Sweden; Karolinska Institutet, Stockholm, Sweden; Karolinska Institutet, Department of Anaesthesia and Intensive Care, Danderyd Hospital, Stockholm, Sweden

## Introduction

Septic shock is associated with high mortality. Elderly and multimorbid patients are not always eligible for intensive care unit (ICU) admission. Intravenous (IV) infusion of norepinephrine (NE) is an accepted treatment for hypotension in septic shock. Use of a vasopressor outside the ICU is poorly evaluated.

## Objectives

To describe the severity of disease using Acute Physiology and Chronic Health Evaluation (APACHE-II) scoring, mortality and adverse events (AE); in patients with septic shock receiving IV fluid and NE in a high dependency unit (HDU) at the Dept of Medicine.

## Methods

A retrospective review of 91 patients with sepsis treated with antibiotics, IV fluids and NE for hypotension. Data on HDU- and 30-day mortality, standardized mortality ratio (SMR) as well as adverse events (necrosis and arrhythmia) were collected. The route of administration of fluids and NE, via peripheral venous catheter (PVC) or central venous catheter (CVC), was registered. All patients were monitored with pulse oximetry, a 3-point ECG and had blood pressure measured non-invasively. Nurse:patient ratio was ≥ 1:3 in the HDU.

## Results

**Figure 1:**
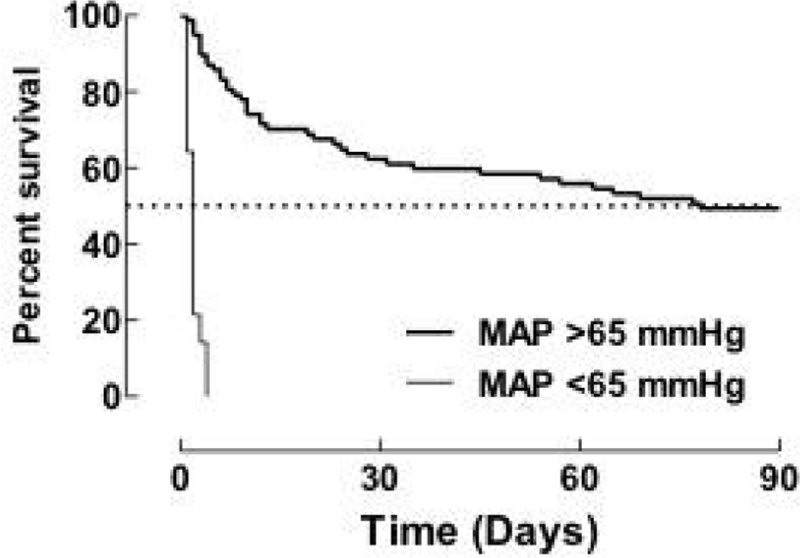
**Patient survival (%)**.

Median age (min-max) was 81 (43-96) years and median APACHE-II was 26 (12-42) points. Patients not reaching a mean arterial pressure (MAP) >65 mm Hg within 12 hours had poor outcome, see Figure 1. HDU-mortality was 27 % (n = 25), with SMR as 0.443 (95 % CI: 0.287-0.654); and 30-day mortality was 47 % (n = 43). NE (maximum 0.2 microgram/kg/min) was administered via PVC in 87 % (n = 79) and via CVC in 13 % (n = 12) of patients, duration up to 72 hours. No skin necrosis was noted; one patient developed sinus tachycardia and died shortly after having the NE-infusion stopped.

## Conclusions

Patients treated with NE for hypotension in septic shock had a better HDU survival than expected. This study does not indicate frequent AEs when administering NE via a PVC.

